# Medial Deviation of a 6° Prosthetic Trochlear Groove After Kinematically Aligned Total Knee Arthroplasty Occurs in Four Types of Coronal Plane Alignment of the Knee (CPAK) and Decreases the Forgotten Joint Score

**DOI:** 10.1016/j.artd.2024.101569

**Published:** 2024-12-12

**Authors:** Dragan V. Jeremic, Johan Bellemans, Elliot Sappey-Marinier, Stephen M. Howell, Werner Hettwer, Maury L. Hull

**Affiliations:** a Clinic for Orthopedic Surgery, St.-Vincenz Hospital, Brakel, Germany; b ArthroClinic Leuven Hasselt University, Hasselt, Limburg, Belgium; c Department of Orthopaedic Surgery, Ramsay Sante, Hôpital Prive Jean Mermoz, Centre Orthopedique Santy, Lyon, France; d Department of Biomedical Engineering, University of California, Davis, CA; e OZB - München, Munich, Germany; f Department of Orthopedic Surgery, University of California Davis Medical Center, Sacramento, CA; g Department of Mechanical Engineering, University of California, Davis, CA

**Keywords:** Total knee replacement, Arthroplasty, Patient outcomes, Patellofemoral joint, Kinematics, Quadriceps vector

## Abstract

**Background:**

The study focused on kinematically aligned total knee arthroplasty (KA TKA). It identified which coronal plane alignment of the knee (CPAK) types are associated with a higher proportion of medial deviation of the 6° prosthetic trochlear groove (PTG) relative to the quadriceps’ line of pull and whether medial deviation adversely affected the Forgotten Joint Score (FJS). The research calculated the minimum PTG angle required to prevent medial deviation by at least 2° in all patients.

**Methods:**

The study analyzed 296 KA TKAs with a postoperative long-leg scanogram and a 2-year FJS score. Radiographic measurements were used to determine the CPAK type and to identify the deviation of the 6° PTG relative to the quadriceps’ line of pull as medial (−) or lateral (+).

**Results:**

Fifty-one percent of KA TKAs had a medial deviation of the PTG, and the proportion was higher in CPAK I, II, III, and VI than in IV and V types (*P* < .05). The median FJS of CPAK III was significantly lower than I and IV (*P* < .0262) and comparable to II, V, and VI (*P* > .085). The minimum PTG angle required to prevent medial deviation by at least 2° in all patients is 17°.

**Conclusions:**

A medial deviation of the 6° PTG occurred in more than half of the KA TKAs and was observed in 4 of 6 CPAK types. Because medial deviation was associated with a lower FJS, it is suggested that the femoral component should have a minimum PTG of 17° to prevent medial deviation by at least 2° in all patients.

**Level of evidence:**

IV.

## Introduction

With the affirmation of international randomized control trials reporting improved knee range of motion and clinical outcome scores [1], kinematic alignment (KA) total knee arthroplasty (TKA) has emerged as a viable alternative to mechanical alignment (MA). In contrast to MA, where all patients receive a neutral limb alignment and joint line obliquity (JLO), which requires collateral ligament release in the majority of patients [2], KA resurfaces the patient's prearthritic knee to restore native limb alignment and knee JLO without collateral ligament release [3].

**Figure 1:**
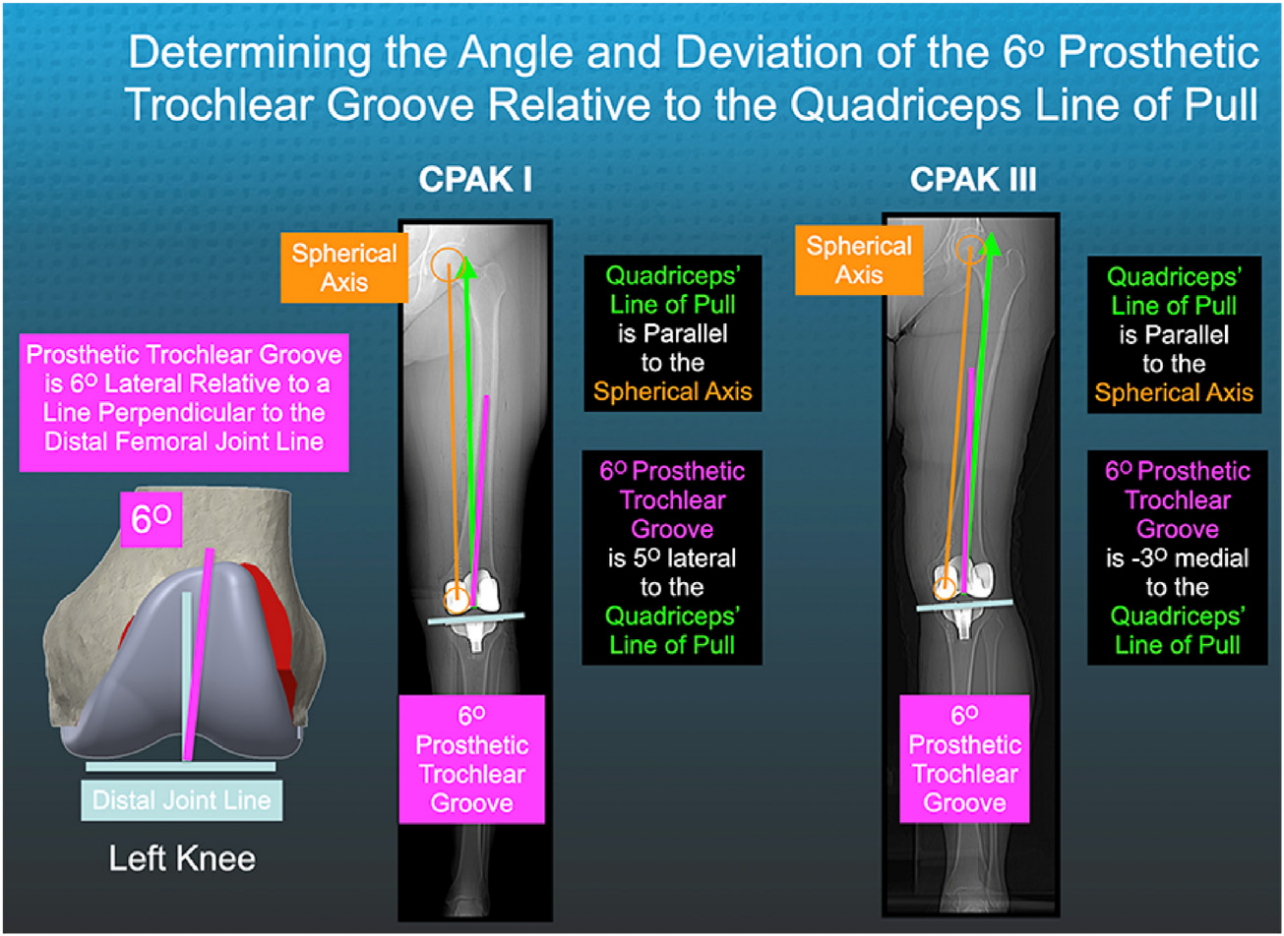
The composite shows a schematic of a left femoral component with a 6° groove (magenta line), a line connecting the deepest points of the prosthetic trochlea, and a long-leg scanogram of a CPAK I and III phenotype depicting the methods for finding the spherical axis (orange line), orienting the quadriceps line of pull (ie, vector) parallel to it (green arrow), and measuring the angle formed by the 6° groove and quadriceps' line of pull. A negative/positive angle indicated medial/lateral deviation of the groove [7].

When surgeons perform KA, they can set the prosthetic trochlear groove (PTG) of a femoral component with an angle of 6° medial or lateral relative to the quadriceps' line of pull (ie, quadriceps vector). Medial deviation of the prosthetic groove can alter patellofemoral kinematics like an incorrectly oriented trochleoplasty and lower the median Forgotten Joint Score (FJS) by 17 points, which exceeds the reported minimum clinically important difference of 14 points [[4], [5], [6]] (Fig. 1). KA of a 6° groove causes medial deviation more frequently than MA because 84% of native distal femoral joint lines are valgus, up to 10°, relative to the femoral mechanical axis [5,8].

A validated, image-based method for constructing the orientation of the quadriceps' line of pull is needed to measure the angle formed by the groove and quadriceps' line of pull and determine whether the groove deviates medial (−) or lateral (+). A principal component analysis using each muscle's direction of pull and volume for force magnitude confirmed that the quadriceps' line of pull is parallel to the spherical axis, a line connecting the centers of the femoral head and the distal medial femoral condyle [7]. Hence, constructing the spherical axis on an anteroposterior (AP) postoperative radiograph of the femur can determine the quadriceps' line of pull.

**Figure 2:**
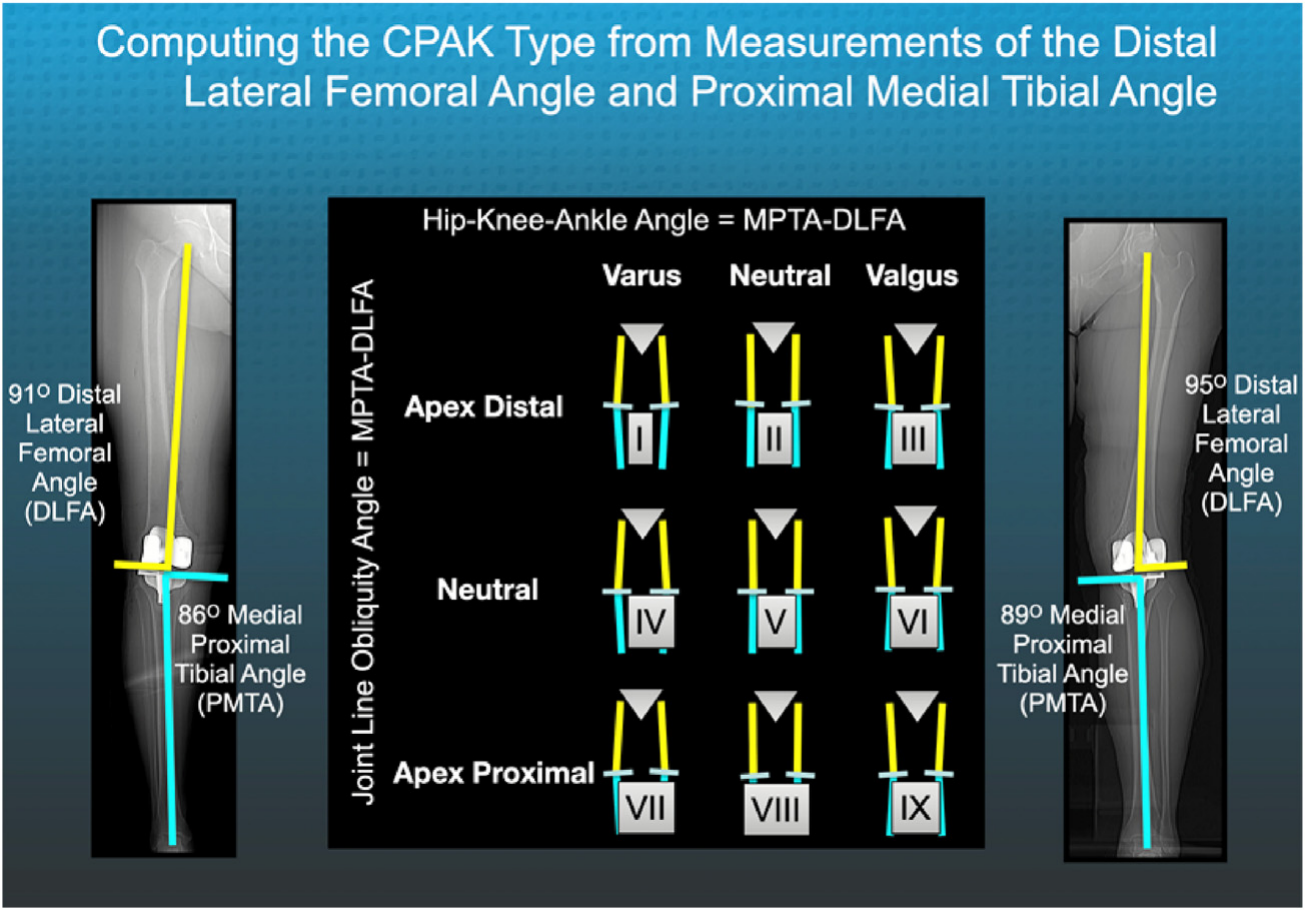
The composite shows a long-leg anteroposterior scanogram of a CPAK I and III depicting the methods for measuring the distal lateral femoral angle (DLFA) and proximal medial tibial angle (PMTA) and computing the limb alignment angle (MPTA-DLFA) and knee joint line obliquity (MPTA + DLFA), which define the 9 types of coronal plane alignment of the knee (CPAK) [10].

It is important to understand which type of limb alignment and JLO, known as coronal plane alignment of the knee (CPAK), is associated with a high risk of medial deviation of a 6° PTG [5,9] (Fig. 2). Identifying the minimum PTG angle required to prevent medial deviation by at least 2° in all patients would be valuable to improve patient satisfaction and those who design femoral components. The minimum groove angle can be calculated by determining from a representative population of patients with KA TKA, the one with the most medial angle of deviation and then adding the angle of the deviation and 2° to the 6° angle of the prosthetic trochlear groove.

Accordingly, the present study examined 296 patients who underwent KA TKA with a 6° PTG and tested 3 hypotheses:

1) Medial deviation of a 6° prosthetic trochlear groove occurs in more than one CPAK type.
2) Patients with medial deviation have lower Forgotten Joint and Oxford Knee scores.
3) There is a groove angle that, if used in all patients, would have prevented medial deviation by at least 2° relative to the quadriceps' line of pull.

## Material and methods

With approval from an institutional review board (Pro00075103), a retrospective analysis of a prospective database identified all patients who underwent a primary KA TKA in 2019 by one surgeon (S.M.H.). Each patient fulfilled the Centers for Medicare & Medicaid Services guidelines for medical necessity for TKA treatment and had (1) Kellgren-Lawrence Grade III to IV osteoarthritis; (2) any severity of varus or valgus deformity; and (3) any severity of flexion contracture. Excluded were patients who had prior fractures of the knee treated with open-reduction internal fixation, inflammatory or septic arthritis, and lower extremity neurologic disorders. Considered for inclusion were 415 KA TKAs.

The surgeon performed a cemented caliper-verified KA TKA using a femoral component designed for MA with a 6° groove, patella resurfacing, and an insert with intermediate medial conformity, flat lateral articular surface, and posterior cruciate ligament retention (GMK Sphere, Medacta International, Castel San Pietro, Switzerland, www.medacta.com, accessed on November 5, 2023). The surgeon used manual instruments and caliper verification to align the components to resurface the patient’s prearthritic knee with a reported accuracy greater than robotics and a negligible learning curve for the inexperienced surgeon [10]. Briefly, for the femoral component, the internal-external (I-E) and varus-valgus (V-V) rotations and the anterior-posterior (A-P) and proximal-distal (P-D) positions were set coincident with the native distal and posterior joint lines by adjusting the calipered thicknesses of the distal and posterior femoral resections to within 0 ± 0.5 mm of those of the femoral component condyles after compensating for cartilage wear and kerf of the saw blade. For the tibial component, the anatomic baseplate should be best fitted parallel to the cortical rim of the tibial resection set to I-E rotation [11]. The knee was balanced by adjusting V-V rotation and medial slope of the tibial resection to match the patient’s prearthritic tibial joint lines and inserting the optimal insert thickness that maximized passive external tibial orientation in extension and internal tibial orientation in 90° flexion as determined by an insert goniometer [12].

On the day of discharge, each patient had an A-P, nonweight-bearing, long-leg scanogram of both legs performed by a computed tomography scanner. The scanogram’s average radiation dose of 0.1 to 0.2 mSv is less than the 0.7 mSv reported for the conventional long-leg radiograph [13]. The technician standardized leg rotation to minimize projection and angular measurement errors by setting the I-E rotation of the knee until each posterior condyle of the femoral component was partially visible on either side of the anterior flange of the femoral component [14].

On the second anniversary of the surgery, digital data collection software (CareSense, Blue Bell, PA, http://caresense.com/, last accessed on November 21, 2023) sent each patient a questionnaire via text or email asking them to fill out the FJS and Oxford Knee Score. One investigator accessed 5 “people search” websites to determine the whereabouts of nonresponding patients, who were contacted by phone when located.

The limbs were assigned to a CPAK type using the methodology described by MacDessi et al [15]. The interobserver was near-perfect (0.99; *P* < .001) for measures between all 4 observers, and the intraobserver (ie, correlation coefficient) was near-perfect (0.99; *P* < .001) for measures within observers at a 1-week interval [15]. An evaluator determined the hip-knee-ankle (HKA) angle by identifying bony landmarks and measuring the medial proximal tibial angle (MPTA) and lateral distal femoral angle (LDFA) and applying the algorithm: HKA angle = MPTA–LDFA using image-analysis software (v.13.0.1 OSIRIX, v.13.0.1, www.osirix-viewer.com last accessed September 30, 2023). The JLO was the sum of the MPTA + LDFA. CPAK describes the direction of the JLO as “apex distal,” “neutral,” and “apex proximal” when the apex of the angle formed by the intersection of the joint lines of both knees at the midline is either below, level with, or above the level of a horizontal joint (Fig. 2). CPAK boundaries for neutral arithmetic HKA angle (aHKAA) are 0° ± 2°. A varus aHKAA is less than −2°, while a valgus aHKAA is greater than +2°. CPAK boundaries for a neutral JLO are 180° ± 3°. An apex distal JLO is less than 177°, while an apex proximal JLO is greater than 183° [15].

One author analyzed the AP scanogram of the femur and measured the angle between the groove, which is a line connecting the deepest points in the prosthetic trochlea, and a line representing the quadriceps’ line of pull centered on the distal intercondylar notch (Fig. 1). A negative/positive angle indicated that the prosthetic trochlear grove deviated medially/laterally relative to the quadriceps line of pull.

### Statistical analysis

Two statistical analyses determined the consistency of measuring the angle between the groove and the quadriceps’ line of pull angle. The first analysis computed the intraobserver and the interobserver variability intraclass correlation coefficient using measurements made on 12 randomly selected TKAs by 3 observers and the variance components for observer, patient, and error determined by a two-factor analysis of variance that modeled observer and patient as random effects [16]. The second analysis quantified repeatability (ie, the precision of measurement) by computing the square root of the pooled variance from 5 measurements of the angle between the groove and the quadriceps’ line of pull made on alternating days on 5 randomly selected TKAs by a single observer. Accordingly, the intraobserver and interobserver intraclass correlation coefficients and precision were 0.96, 0.95, and 0.24°, respectively. The reported intraobserver and interobserver intraclass correlation coefficients are 0.96 and 0.95, respectively, and the precision of the difference is 0.2°.

The sample size was calculated using a one-way analysis of variance (G∗Power access date: 21 November 2023). The independent variable was the CPAK type at 6 levels (ie, CPAK I–VI). The FJS was the primary dependent variable because it detects differences between patients when the Oxford Knee Score cannot because of the higher ceiling effect [17]. With an α = 0.05, a power = 0.80, a FJS of 14, as it is the minimum clinically important difference, and a standard deviation of 20, the smallest sample size was 32 patients per CPAK category [6].

The authors used statistical software (JMP Pro, 17.2.0, http://www.jmp.com, access date: 21 November 2023), with *P* < .05 indicating a significant difference. The mean ± standard deviation and median and interquartile range described normal and non-normal random variables, respectively. The Fisher's exact test determined whether the medial and lateral deviation proportions of the 6° groove differed across all 6 CPAK types and which pairs of CPAK types were different. Because the Kruskal-Wallis H test was nonrobust due to a wide disparity in the sample size between the CPAK types that ranged from 6 to 143 subjects, the Wilcoxon rank-sum test determined whether the Forgotten Joint and Oxford Knee scores differed between CPAK types.

## Results

**Figure 3:**
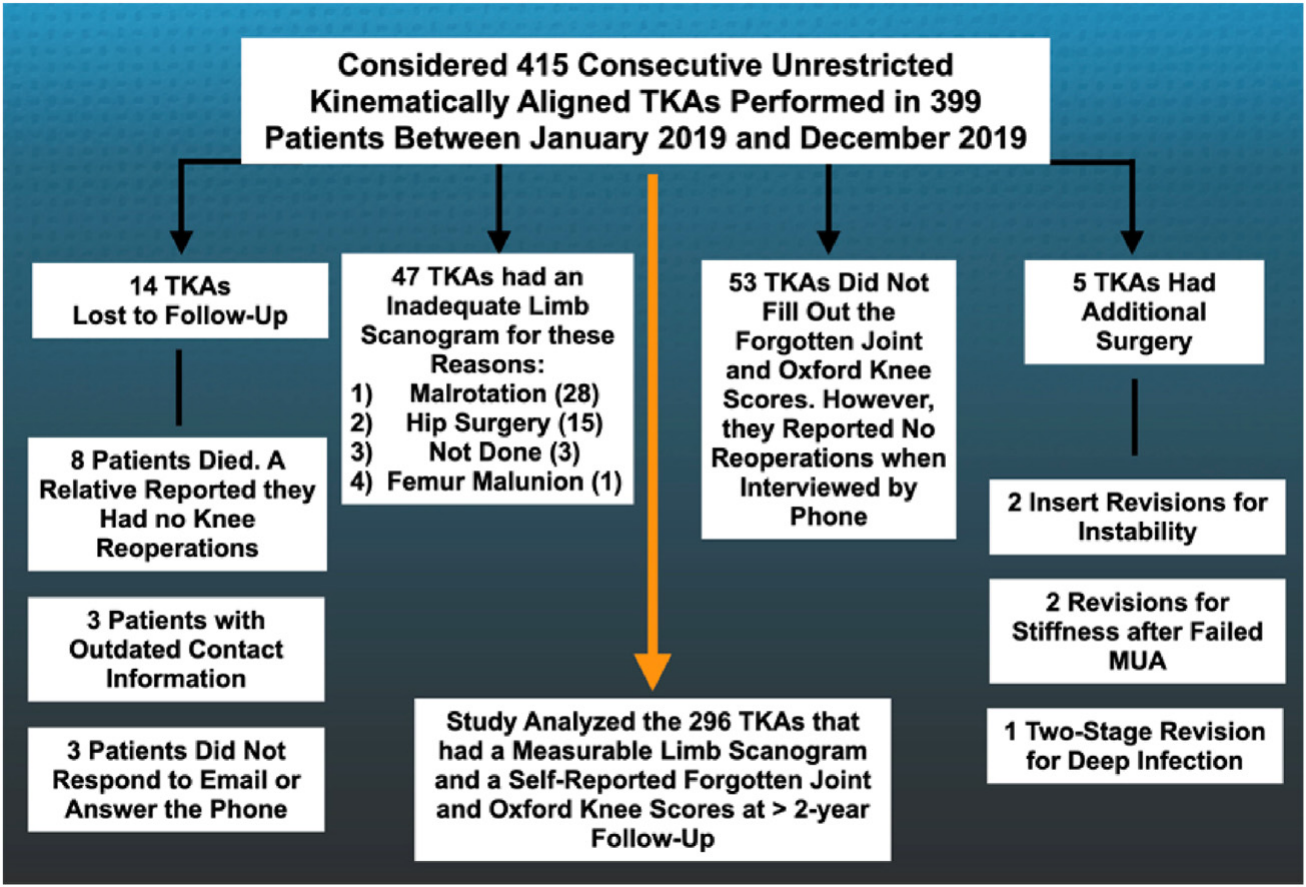
The flow diagram shows the reasons for excluding 119 KA TKAs from an initial cohort of 415 KA TKAs (399 patients) considered eligible for the study.

The Consolidated Standards of Reporting Trials diagram illustrates the reasons for excluding 119 KA TKAs from an initial cohort of 415 KA TKAs (399 patients) considered eligible for the study (Fig. 3). The 4 reasons were: 1) loss to follow-up (n = 14); 2) inadequate limb scanogram (n = 47); 3) incomplete FJS and Oxford Knee Score questionnaires (n = 53); and 4) additional knee surgery (n = 5). Consequently, 296 KA TKAs were analyzed. Table 1 compares patient demographics and preoperative characteristics, such as sex, body mass index, maximum knee flexion, and clinical scores, for the included and excluded patients with KA TKAs. A contingency analysis revealed no significant difference in the proportion of the 296 included KA TKAs and 66 excluded KA TKAs with a readable AP long-leg scanogram within each CPAK category (*P* = .1339).

**Table 1:** The preoperative characteristics and function scores for the included and excluded TKAs in the present study and the results of the statistical analysis of the differences between groups.

Preoperative characteristics	Included subjects^a^	Excluded subjects^a^	*P*-value
Number of KA TKAs	296	119	
Age (y)	69 ± 8 (35-88)	70 ± 8 (49-91)	.168
Sex	157 females, 126 males	69 females, 45 males	.368
Body mass index (kg/m^2^)	30 ± 5 (23-47)	30 ± 6 (23-47)	.135
Knee range of motion (º)	105 ± 10 (60-130)	101 ± 12 (71-124)	.006
Knee Society Score Knee (100 is best, 0 is worst)	37 ± 13 (0-88)	36 ± 12 (4-66)	.855
Knee Society Score Function (100 is best, 0 is worst)	55 ± 20 (0-100)	48 ± 16 (0-100)	.002
Oxford Knee Score (48 is best, 0 is worst)	23 ± 8 (5-46)	21 ± 8 (0-41)	.023
Knee Injury and Osteoarthritis Outcome Score for Joint Replacement (KOOS JR) (100 is best, 0 is worst)	49 ± 14 (31-100)	46 ± 13 (8-92)	.026

a Values reported are the mean ± standard deviation (range).

**Figure 4:**
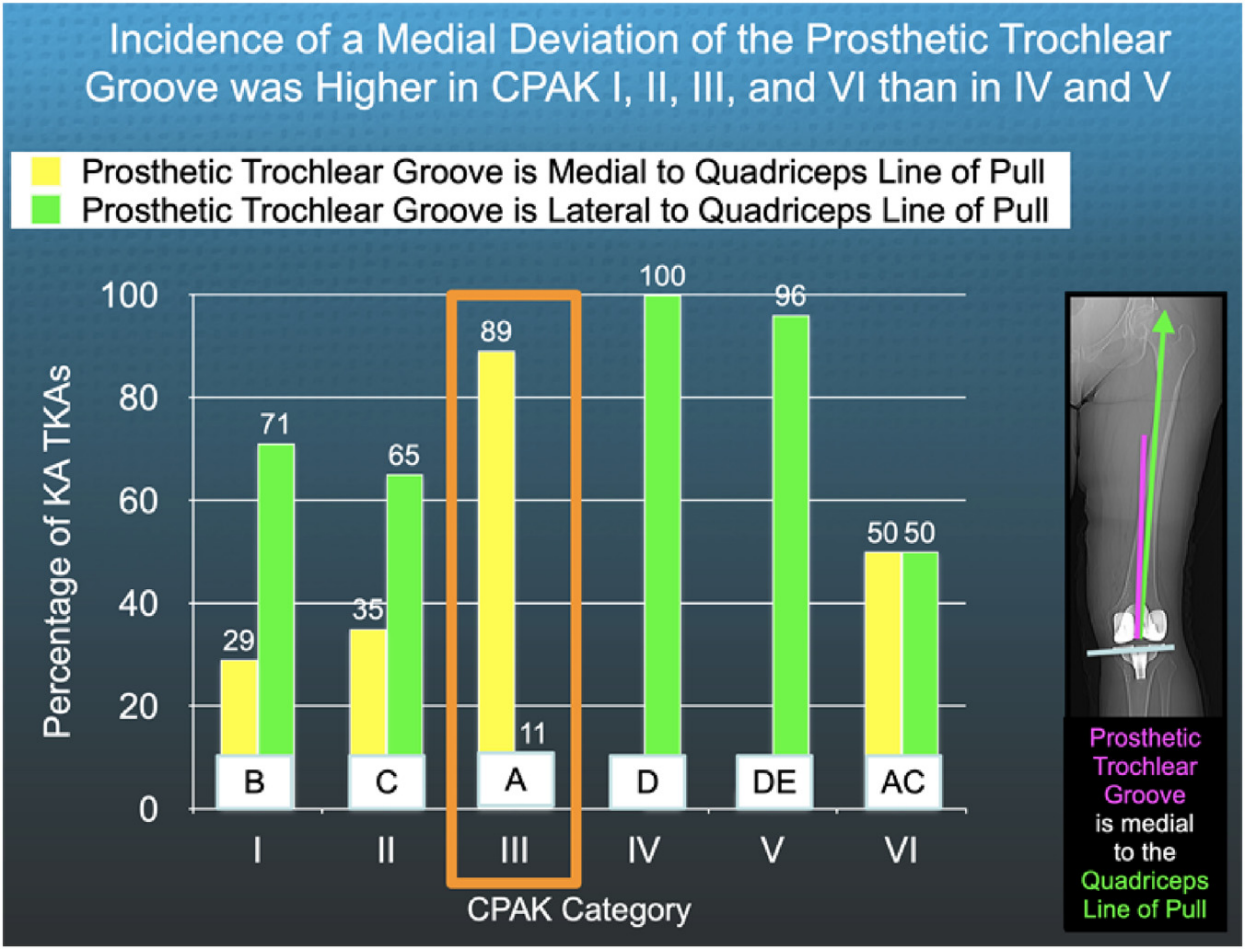
The column graph shows, for each CPAK category, the percentage of KA TKAs where the prosthetic trochlear groove deviated medially and laterally relative to the quadriceps line of pull. CPAK III had the highest incidence of a medial deviation of 89% (orange rectangle). The percentages in categories denoted by different capital letters are significantly different.

**Figure 5:**
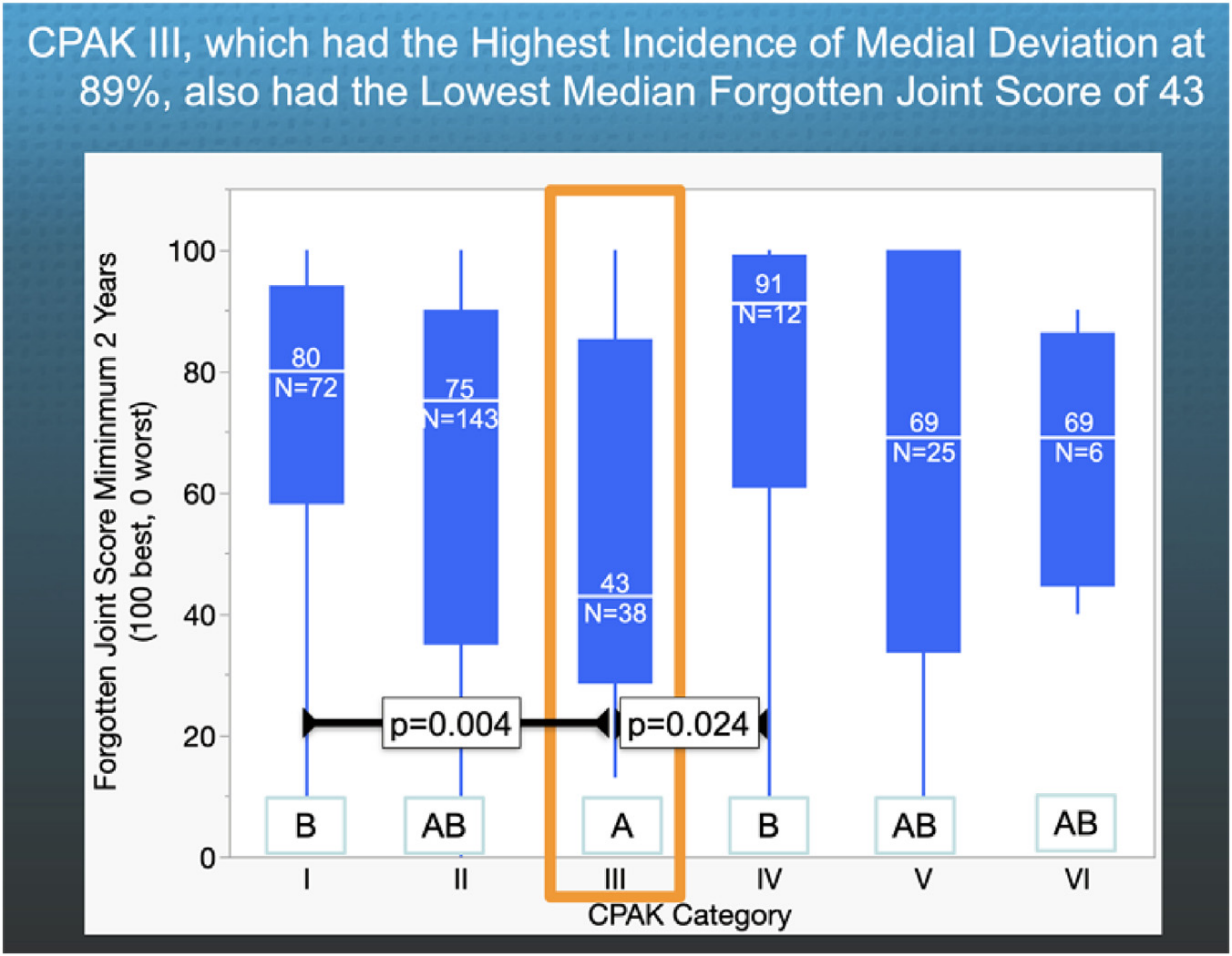
The quantile box plot shows the number of KA TKAs and the median Forgotten Joint Score (transverse white line within a blue rectangle) for each CPAK category. Those medians denoted by different capital letters are significantly different. The CPAK III score of 43 was significantly lower than the 80 and 91 scores for varus limb CPAK I and IV and exceeded the minimum clinically important difference of 14 points (*P* ≤ .0237).

**Figure 6:**
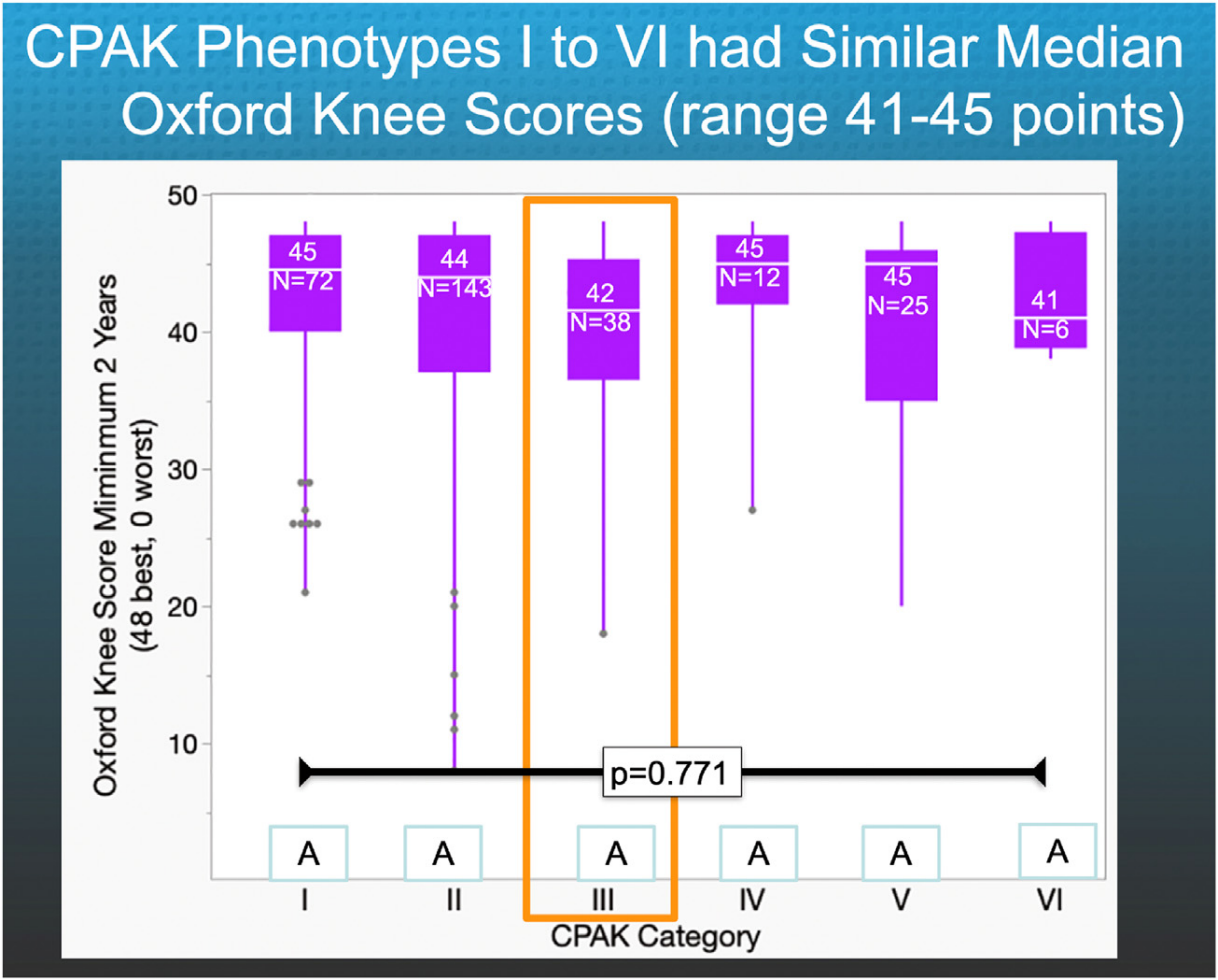
The quantile box plot shows the number of KA TKAs and the median Oxford Knee Score (transverse white line within a purple rectangle) for each CPAK category. The median did not differ between CPAK I to VI (*P* = .7710) because many subjects had a maximum score of 48, which created a ceiling effect.

The following are the relevant results. Fifty-one percent of KA TKAs had a medial deviation of the PTG. The proportion of KA TKAs with medial deviation was higher in CPAK I (29%), II (35%), III (89%), and VI (50%) than in IV (0%) and V (4%) patients (*P* < .05) (Fig. 4). CPAK III, which had the highest incidence of medial deviation at 89%, also had the lowest median Forgotten Joint Score of 43. The median Forgotten Joint Score was significantly lower in CPAK III (43) than in CPAK I (80) (*P* = .0068) and IV (90) (*P* = .0262), and comparable to CPAK groups II (75), V (69), and VI (69) (*P* > .089) (Fig. 5). The median Oxford Knee Score did not differ between CPAK types (*P* = .7710) (Fig. 6). The minimum prosthetic groove angle required to prevent medial deviation by at least 2° in all patients is 17°.

## Discussion

The study revealed three main findings: 1) Medial deviation was observed in 51% of knee arthroplasty surgeries across CPAK types I, II, III, and VI; 2) CPAK III, which had the highest incidence of medial deviation at 89%, also had the lowest median Forgotten Joint Score of 43 points; and 3) a 17° prosthetic groove would have prevented medial deviation by at least 2° in all patients in this study.

The present study showed that a 6° groove lowered the FJS, and a lower score is likely to occur when most currently available femoral components designed explicitly for MA are used when performing KA TKA [5]. A study of 34 MA-designed femoral components reported that the PTG, which averaged 6°, is too restrictive as it was too narrow to accommodate the up to 30° groove of 4113 native trochlear [4]. Hence, the PTG should be more lateral than 6°, consistent with the present study’s finding that 17° is the minimum groove angle that would have prevented medial deviation by at least 2° in all patients [4,5,18].

One option to possibly improve the FJS is to use a femoral component explicitly designed for KA to eliminate the risk of medial deviation relative to the quadriceps’ line of pull. One design requirement is to increase the groove angle to lateralize the proximal opening of the PTG, using as a reference, the minimum angle of 17° reported in the present study. A three-dimensional modeling study of a KA-designed femoral component with a 20° groove reported it had the potential to mitigate the risk of patellofemoral complications by lateralizing and widening the groove to avoid medializing the patella for wide variations in the lateral distal femoral angle [19]. A recent radiologic study of sequential bilateral KA TKA in 36 patients with a KA-designed femoral component with a 20° groove in one knee and an MA-designed femoral component with a 6° groove in the other confirmed that the 20° groove was lateral to the quadriceps’ line of pull in all knees [18].

A second design requirement is to extend the anterior flange laterally to cover the lateral side of the anterior femoral resection and better funnel patella engagement. The need for better coverage is consistent with several reports. One radiographic study measured under coverage of the lateral anterior femoral resection in patients with a valgus distal lateral femoral joint line after performing KA TKA with a prosthetic trochlear angle ranging from 6° to 8.3° (Gemini MK II, Link, Hamburger, Germany) [20]. A three-dimensional modeling study using a KA-designed femoral component with a more lateralized prosthetic trochlea than an MA-design reduced lateral under coverage of the flange from 4 mm to 2 mm [19], a finding supported in a radiographic study using a KA-designed femoral component with a 20° groove [18].

In patients with CPAK III, it might be hypothesized to consider using mechanical alignment when only a medialized 6° prosthetic groove is available instead of kinematic alignment, especially if the surgeon suspects that dissatisfaction in primary knee replacements for valgus osteoarthritis is due to pain from excessive medial collateral ligament strain, which can be managed by changing the constitutional pre-existing valgus limb alignment to an abnormal 0° HKA angle [21]. A post-hoc analysis tested this hypothesis by categorizing the limb alignment of the KA TKAs into groups based on the degree of postoperative valgus alignment. It was found that the 88-point median FJS of the subjects with ≥5° valgus was significantly higher than the 43-point score for the 3-4° valgus group (*P* = .0414), which disproved the hypothesis. This indicates the lower scores in CPAK III are not explained by excessive medial collateral ligament strain from restoring the constitutional valgus of the prearthritic limb. It is important to note that changing the prearthritic valgus limb alignment to an abnormal 0° HKA angle may not necessarily lead to better outcomes. A 1 mm or greater overresection of the distal medial femur relative to the prearthritic articular surface, which occurs in 85% of mechanically aligned TKAs and nearly all valgus limbs, causes a 35-point reduction in the FJS to a median value of 40 points, which is not any better than the 43-point score for the KA TKAs in the CPAK III category in the present study [22].

Several limitations could affect the generalization of the study’s results. First, the method for defining the orientation of the quadriceps’ line of pull on an AP radiograph of the femur is an approximation for the actual quadriceps line of pull because it does not account for muscle volume and, hence, force variations between patients. However, the error caused by muscular force variations between patients was small, as it did not prevent the identification of those patients with a medial deviation of the PTG and the association with a lowering of the FJS that exceeded 14 points, the reported minimum clinically important difference [6]. Second, medial deviation might not be a predictor of a lower FJS with other alignment strategies, such as mechanical, functional, and restricted KA, because they can change the prearthritic femoral and tibial articular surfaces, limb alignment, and joint line [[23], [24], [25], [26]]. Changing the prearthritic distal and posterior medial femur by an overresection of bone as little as 1 mm lowered the FJS by 35 and 40 points, respectively, 2 times more than the medial deviation of the groove did in the present study [23]. Finally, the omission of listing the radiographic indicators of patellofemoral tracking, including the patella tilt angle, lateral patella translation, and lateral undercoverage of the anterior femoral resection, did not affect the conclusion of the study because, in KA TKA, they do not predict clinical outcome scores and dissatisfaction [27].

## Conclusions

Surgeons that follow the guidelines and surgical principles of KA TKA resurface the prearthritic knee without referencing the center of the femoral head and ankle. They, therefore, do not obtain a preoperative long-leg radiograph, which means they do not know whether the patient has medial or lateral deviation of the 6° PTG. Surgeons that obtain a preoperative long-leg radiograph must construct lines along the PTG and quadriceps line of pull to determine medial or lateral deviation, as patients with medial or lateral deviation exist in 4 of 6 CPAKs. A strategy for potentially improving the FJS in over half of the KA TKAs with medial deviation is to use a femoral component with a minimum PTG of 17° would have prevented medial deviation by at least 2° in all patients. Lateralizing the PTG from 6° to 17° provides a broader funnel to capture the patella. It does not change the medial geometry of the trochlea, which is responsible for the medial containment of the patella. Further study is needed to determine whether a 17° prosthetic trochlear groove has any negative clinical effects.

## Conflicts of interest

D. V. Jeremic is a paid consultant for Medacta. J. Bellemans is a paid consultant and receives royalties from Medacta and Smith and Nephew. M. L. Hull receives research support from Medacta. S. M. Howell is a paid consultant and receives royalties from Medacta. All other authors declare no potential conflicts of interest.

For full disclosure statements refer to https://doi.org/10.1016/j.artd.2024.101569.

## CRediT authorship contribution statement

**Dragan V. Jeremic:** Writing – review & editing, Writing – original draft, Methodology, Conceptualization. **Johan Bellemans:** Writing – review & editing, Writing – original draft, Conceptualization. **Elliot Sappey-Marinier:** Writing – review & editing, Writing – original draft, Methodology, Data curation, Conceptualization. **Stephen M. Howell:** Writing – review & editing, Writing – original draft, Methodology, Investigation, Formal analysis, Conceptualization. **Werner Hettwer:** Writing – review & editing, Writing – original draft, Conceptualization. **Maury L. Hull:** Writing – review & editing, Writing – original draft, Methodology.

## Appendix A Supplementary data

Conflict of Interest Statement for Jeremic

Conflict of Interest Statement for Sappey-Marinier

Conflict of Interest Statement for Bellemans

Conflict of Interest Statement for Hull

Conflict of Interest Statement for Howell

Conflict of Interest Statement for Hettwer
