# Supplementary material for: Medial Deviation of a 6° Prosthetic Trochlear Groove After Kinematically Aligned Total Knee Arthroplasty Occurs in Four Types of Coronal Plane Alignment of the Knee (CPAK) and Decreases the Forgotten Joint Score
Source: Arthroplast Today. 2024 Dec 12;30:101569. doi: 10.1016/j.artd.2024.101569 (PMC11699096; doi:10.1016/j.artd.2024.101569)
Supplement: Conflict of Interest Statement for Jeremic [file mmc1.docx]

# INDIVIDUAL CONFLICT OF INTEREST STATEMENT

***American Association of Hip and Knee Surgeons***

(Adopted from the American Academy of Orthopaedic Surgeons disclosure statement)

The following form **must be filled out completely and submitted by each author (example, 6 authors, 6 forms).**

**All items require a response. If there is no relevant disclosure for a given item, enter "*None*.”**

**Similar Coronal Plane Alignment of the Knee (CPAK) in Unrestricted Kinematically Aligned TKA and the Native Limb and a Potential to Improve the Forgotten Joint Score in CPAK III**

**Manuscript Title**

1. Royalties from a company or supplier (The following conflicts were disclosed)

**None**

2. Speakers bureau/paid presentations for a company or supplier (The following conflicts were disclosed)

**None**

3A. Paid employee for a company or supplier (The following conflicts were disclosed)

**None**

3B. Paid consultant for a company or supplier (The following conflicts were disclosed)

**Medacta**

3C. Unpaid consultants for a company or supplier (The following conflicts were disclosed)

**None**

4. Stock or stock options in a company or supplier (The following conflicts were disclosed)

**None**

5. Research support from a company or supplier as a Principal Investigator (The following conflicts were disclosed)

**None**

6. Other financial or material support from a company or supplier (The following conflicts were disclosed)

**None**

7. Royalties, financial or material support from publishers (The following conflicts were disclosed)

**None**

8. Medical/Orthopaedic publications editorial/governing board (The following conflicts were disclosed)

**None**

9. Board member/committee appointments for a society (The following conflicts were disclosed)

**None**

**Each author must sign AND print or type his/her name, date and submit a separate form**

In addition, one BLINDED Conflict of Interest form (no author names used) should be submitted per manuscript with all author disclosures.


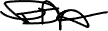


JEREMIC Dragan 12/10/2022

Author Name (Print or Type) Author Signature Date
